# Distribution Characteristics and Controlling Factors of Soil Total Nitrogen: Phosphorus Ratio Across the Northeast Tibetan Plateau Shrublands

**DOI:** 10.3389/fpls.2022.825817

**Published:** 2022-04-12

**Authors:** Xiuqing Nie, Dong Wang, Lining Ren, Kaili Ma, Yongzhe Chen, Lucun Yang, Yangong Du, Guoying Zhou

**Affiliations:** ^1^Key Laboratory of Forest Ecology and Environment of National Forestry and Grassland Administration, Ecology and Nature Conservation Institute, Chinese Academy of Forestry, Beijing, China; ^2^Northwest Institute of Plateau Biology, Chinese Academy of Sciences, Xining, China; ^3^Research Institute of Forestry, Chinese Academy of Forestry, Beijing, China; ^4^Monitoring and Evaluation Center of Qinghai National Park, Xining, China

**Keywords:** soil N:P ratio, tibetan plateau, shrublands ecosystem, controlling factors, distribution, alpine zone

## Abstract

Nitrogen (N) and phosphorus (P) stoichiometry have significant effects on nutrient cycles in terrestrial ecosystems. However, our understanding of the patterns and the driving factors of soil N:P ratios in the Tibetan Plateau shrublands remains limited. Our study aimed to quantify the distribution of soil N:P ratio and its controlling factors based on soil, plant, and climate factors from 59 sites in shrublands across the northeast Tibetan Plateau. The kriging interpolation method was used to quantify the soil N:P distribution. Spatially, the soil N:P ratio was higher in the south than in the north and lower in the west than in the east. The soil N:P ratio in the northeast Tibetan Plateau shrublands was mainly explained by edaphic factors, which also played an important role in regulating the effects of plant and climate factors on soil N:P ratios. Mean annual precipitation, instead of mean annual temperature, significantly controlled the soil N:P ratios, and its effect on the pattern of soil N:P ratios differed between alpine shrublands and desert shrublands. The N:P ratios of different organs in shrublands also played different roles in shaping the soil N:P ratios in alpine and desert shrublands. These results provide support for the hypothesis that edaphic factors were the dominant drivers of spatial variation in soil N:P ratios across the northeast Tibetan Plateau shrublands, and our study contributes to a deeper understanding of biogeochemical cycling at high altitudes.

## Introduction

Nitrogen (N) and phosphorus (P), key components of biomacromolecules in all organisms (Vrede et al., 2004), are not only the most crucial nutrients for plant growth, and their stoichiometry, but can also regulate most ecological processes, such as respiration and decomposition in terrestrial ecosystems (Aerts and Chapin, 2000; Sistla and Schimel, 2012). In the scenario of atmospheric deposition and fertilization, terrestrial ecosystems have been experiencing unprecedented nutrient inputs, including those of N and P (Tipping et al., 2014). The unbalanced N and P inputs can critically affect organisms and soil stoichiometry, which can alter ecosystem functioning (Peñuelas et al., 2015). The biogeochemical cycles of N and P are biologically coupled from molecular to global scales (Finzi et al., 2011). Because P and N cycles interact closely, individual cycles of N and P elements in isolation are potentially not sensitive to ecological functions (Zhang et al., 2019b).

Ecological stoichiometry, such as soil stoichiometry, provides a framework for exploring and predicting global change effects at various scales (Zechmeister-Boltenstern et al., 2015). Furthermore, the identification of spatial patterns in soil N and P stoichiometry can improve our understanding of the dynamics of nutrient cycles in terrestrial ecosystems and their effects on ecosystem functions in a rapidly changing environment (Chen et al., 2016b). The N and P stoichiometry characteristics in grasslands (Jiao et al., 2016), farmlands (Muhammed et al., 2018), forests (Chen et al., 2016b), and desert ecosystems (Zhang et al., 2019a) have been explored, and it was discovered that its characteristics and controlling factors are significantly related to ecosystems (Li et al., 2020; Luo et al., 2020), due to spatial heterogeneity in local soils, plants, and weather conditions in various landscapes (Hobbie et al., 2002). In addition, some studies on soil stoichiometry have been conducted in shrubland ecosystems, such as the regions of the basins of China (Li et al., 2020). On the Tibetan Plateau, soil stoichiometry has been explored in alpine steppes, meadows, deserts, and swamp meadows (Tian et al., 2018), and their stoichiometry varies significantly among different vegetation types (Tian et al., 2018). Although the soil N:P ratio on the Tibetan Plateau generally was 5.9 ± 0.76 (Tian et al., 2010), the soil N:P ratio for the Tibetan Plateau shrublands was unclear.

It has been suggested that the N and P cycles could be uncoupled in the scenario of rapid climate change, owing to the different degrees of control exerted on the supply of these elements by geochemical and biological processes (Delgado-Baquerizo et al., 2013). However, conclusions about the relationship between soil N:P ratio and climatic factors are contrasting in different studies. Specifically, although soil N:P ratio in woodlands and grasslands had a significant increasing trend with mean annual precipitation (MAP) on a regional scale (Li et al., 2020), other studies have indicated that the soil N:P ratios had a negative relationship with precipitation in both forests and grasslands (Chen et al., 2016b; Jiao et al., 2016). Similar to climate factors, changes in soil pH can significantly affect soil N (Nie et al., 2017), which in turn inevitably influences the soil N:P ratio. It has been demonstrated that soil pH was significant in shaping soil N:P ratios in the drylands of northern China (Wang et al., 2020), and was even the most robust factor controlling soil N:P ratios in mature subtropical broadleaf forests (Qiao et al., 2020), and grasslands in the Inner Mongolian Plateau (Jiao et al., 2016). Although there is substantial variation in soil pH (Yang et al., 2015), soil pH affects soil N:P ratios in the high-altitude areas of the Tibetan Plateau, and this effect has been largely unreported. Variations of soil stoichiometry are driven not only by climate factors and soil texture (Wang et al., 2020). It has also been demonstrated that stoichiometry in plant organs can significantly affect the soil N:P ratios in subtropical forests (Fan et al., 2015; Luo et al., 2020). Plants are crucial for driving inputs and retention of nutrient elements; therefore, soil stoichiometry dramatically changes due to various plant species (Yimer et al., 2006; Chen et al., 2016a). However, the roles of plant, climate, and edaphic factors in shaping the soil N:P ratio in shrublands still remain unclear.

Controlling factors related to climate, edaphic characteristics, and plant characteristics can shape soil stoichiometry (Chen et al., 2016b). However, there have been relatively few studies on the distribution and controlling factors of the soil N:P ratios across the Tibetan Plateau shrublands. Furthermore, as the highest altitude and largest plateau in the world (Yao et al., 2017), the biophysical processes on the Tibetan Plateau may differ from other regions (Yang et al., 2008; Chen et al., 2016c). We aimed to answer the following questions: (1) how are shrubland soil N, P, and N:P ratios distributed along controlling factors? (2) how vegetation, climate, and soil characteristics shape the soil N:P ratios and based on this, we quantified the most influential factor on the soil N:P ratios in the northeast Tibetan Plateau shrublands. We hypothesized that soil characteristics are the factor that most significantly controls the soil N:P ratio. Although climatic factors and plant characteristics can, to some extent, affect the inputs and outputs of soil N and P (Chen et al., 2016b), soil characteristics may be more significant because soil N is mainly from soil organic matter (Nie et al., 2020), and soil P is also partly derived from the decomposition of organic matter (Delgado-Baquerizo et al., 2013).

## Materials and Methods

### Study Regions

**FIGURE 1:**
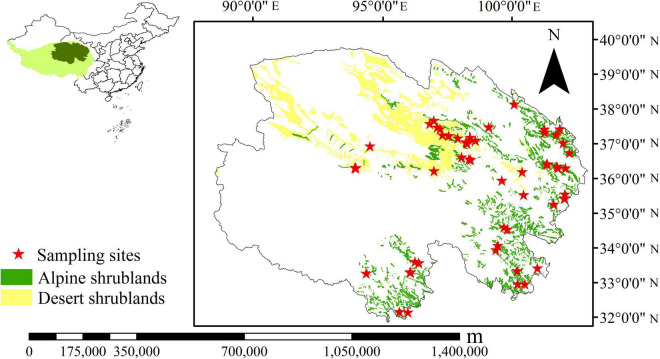
Distribution of sampling sites on the northeast Tibetan Plateau (Chinese Academy of Sciences, 2001).

This study surveyed alpine shrublands and desert shrublands across the northeast Tibetan Plateau (Figure 1). Shrublands consist of woody plants, with a mean height of fewer than 5 m and more than 30% coverage (Wu, 1980). Alpine shrublands and desert shrublands have been primarily classified in the study area (Zhou et al., 1987), and their area is 4.29 × 10^4^ and 6.69 × 10^4^ km^2^, respectively (Chinese Academy of Sciences, 2001). Desert shrublands are mostly distributed in drier regions and comprise plants that can survive in severe drought environments, such as *Sympegma ragelii* and *Kalidium foliatum.* Thus, in desert shrublands, only a few super-xerophytic herbs can endure. The soil types in these regions are mostly gray-brown desert soil and brown desert soil (Zhou et al., 1987). There are 18 sampling sites for desert shrublands. In contrast, alpine shrublands are distributed in the mountains in semiarid and cold environments, and soil types mainly include alpine shrubby meadow soil and chestnut soil (Zhou et al., 1987). Representative plants include *Sibiraea laevigata*, *Rhododendron capitatum*, and *Rhododendron thymifolium*. Herbs, such as *Kobresia* sp., *Carex* sp., and *Oxytropis* sp., are found in alpine shrublands. There are 41 sampling sites for alpine shrublands. Both alpine shrublands and desert shrublands can contribute to soil and water conservation, which are important for the local environment. In alpine shrublands, some herbage, i.e., *Kobresia*, *Stipa aliena*, *Poa pratensis*, are high-quality forage for Tibetan yak and horse. In desert shrublands, different types of vegetation not only play an important role in desertification but also can be used as medicine, i.e., *Ephedra przewalskii*. The range of the altitude for sampling sites was between 2,738 and 4,296 m. Mean annual temperature (MAT) and MAP range from −5.6°C to 8.9°C and 17.6 to 764.4 mm, respectively (Zhang, 2009). Meanwhile, the climate of the Tibetan Plateau has been significantly changing in the past decades; specifically, the MAT and MAP increased by 0.05°C every year, and 10.2 mm every decade, respectively (Yang, 2018).

### Samples and Analysis

To estimate the distribution pattern of soil N:P ratio, 177 soil profiles at 59 sites were investigated in the growing season of July and August from 2011 to 2013. Chinese Academy of Sciencess was funded by the Chinese government to implement a 5-year Strategic Priority Project of Carbon Budget by using about US$35 million. The project was conducted consistently, and specific methods were introduced in the Technical manual writing group of ecosytem carbon sequestration project (2015). These selected sites should be more than 1 ha, which should meet the following characteristics, such as the relatively uniform distribution of community structure, habitat, and species composition. At each site, one plot was excavated, and its length, width, and height were 1, 1.5, and 1 m, respectively. Three soil profiles, namely, the left, right, and front profiles, were excavated in each plot. Soils at depths of 0–10, 10–20, 20–30, 30–50, 50–70, and 70–100 cm were sampled from three profiles at the same depth, and three soil samples were mixed into one. The fine roots were eliminated before soil samples were ground using a ball mill before element measurement (Technical manual writing group of ecosytem carbon sequestration project, 2015). Three 5 m × 5 m plots and 10 m × 10 m plots were selected at each site to represent the natural alpine shrubland and desert shrubland communities, respectively. In each plot, the biomass of fully expanded foliage, stems, and roots of dominant shrubland species was harvested, and subsequently, plant samples were oven-dried and ground after being transported to the laboratory. Soil organic carbon (SOC) was estimated by wet oxidation using the Walkley–Black method (Conyers et al., 2011; Nie et al., 2019). The total N in soil and N content in plant samples (foliage, stem, and root) were analyzed using dry combustion with an elemental analyzer (2400 II CHNS/O, Perkin-Elmer, United States), and the combustion and reduction temperatures were set to 950°C and 640°C, respectively (Wang et al., 2019). The total P content in soil and P content in plant samples (foliage, stem, and root) were measured by the molybdate/ascorbic acid method with H_2_SO_4_-H_2_O_2_ digestion (Jones, 2001). The PHS-3C meter was employed to measure soil pH with a 1:2.5 soil:water mixture. One site was mainly dominated by *Caragana tibetica*, belonging to *Leguminosae* sp., and its response indicators, such as root N:P ratio, were significantly larger than those of other samples. Hence, the results were not considered in the following statistical analyses. To explore the effects of climate factors on soil N:P ratio, climatic data for sampling sites were calculated from the meaning of 1950–2000 from the global climate data^1^ using geographical coordinates, and a spatial resolution was 1 × 1 km (Hijmans et al., 2005).

### Statistical Analysis

Kriging interpolation method was used to interpolate the value of a target variable for unobserved locations using observations at nearby locations (Krige, 1951). The method has been viewed as a practical method for spatial prediction from sit-level upscale to the whole study area (Yang et al., 2010; Nie et al., 2019). ANOVA was conducted to explore whether the soil N and P content and their ratio of desert shrublands and alpine shrublands differed significantly in different soil depths (*P* < 0.05). Ordinary least squares regression was employed to explore the relationships between soil N:P ratio, MAT, SOC, MAP, soil pH, and organs, including stem, root, and foliage N:P ratios, except the relationship between stem N:P ratio and soil N:P ratio, which was presented by curve fitting of the power function. Multivariate regression analysis was employed to explore the effects of edaphic factors, including soil pH, soil N content, and SOC. The analysis was undertaken using the R software package (R Development Core Team, 2012).

The independent effects from the soil, climate, and plant properties on the soil N:P ratios were explored using the method of partial correlation by controlling for other factors. Partial correlation analysis can control effects from a specific factor on the relationship between the response variable and other predictors (Doetterl et al., 2018).

The factors that have significant relationships with the soil N:P ratios were used to conduct subsequent variation partitioning analysis. Soil N:P ratio variation in the alpine shrublands was decomposed by three explanatory variables, including soil, plant, and climate properties, and their joint effects. Soil properties include soil N, SOC, and soil pH. The vegetation properties refer to the stem N:P ratios and root N:P ratios in alpine shrublands, and root N:P ratios in desert shrublands and shrubland types in all shrublands. Variation partitioning analysis can estimate the amount of explained variation for response variables by the chosen factors (Legendre, 2008). The “VEGAN” package was used to conduct this analysis in R software (R Development Core Team, 2012).

Structural equation modeling was employed to determine the relative importance of direct and indirect pathways of driving factors regulating the soil N:P ratio in shrublands. An *a priori* conceptual model was conducted before analysis of the structural equation model (Supplementary Figure 1). The MAP, soil pH, SOC, root N:P ratio, and soil total nitrogen (STN) were chosen for structural equation modeling due to their significant relationship with the soil N:P ratio. Theoretically, lots of studies have assumed that plant, climate, and edaphic factors significantly influence soil stoichiometry, and we hypothesized that MAP and root N:P ratio directly influenced soil N:P ratios, and also indirectly *via* soil pH, SOC, and STN (Supplementary Figure 1). Soil N:P ratios, MAP, soil pH, SOC, root N:P ratio, and STN were scaled to mean = 0 and SD = 1. The value of Fish’s C was statistically non-significant when a good model fit was necessary (Lefcheck, 2015). The Akaike information criterion and path coefficient values were also shown. The “PIECEWISE” package was employed in the statistical R software (R Development Core Team, 2012).

## Results

### Spatial Patterns of Soil N:P Stoichiometry and Its Controlling Factors

**FIGURE 2:**
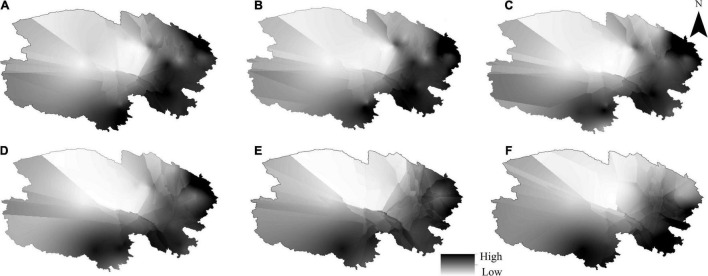
Spatial distribution of soil nitrogen: phosphorus (N:P) ratio in soil depths of 0–10 cm **(A)**, 10–20 cm **(B)**, 20–30 cm **(C)**, 30–50 cm **(D)**, 50–70 cm **(E)**, and 70–100 cm **(F)** in the shrublands on the Tibetan Plateau.

**FIGURE 3:**
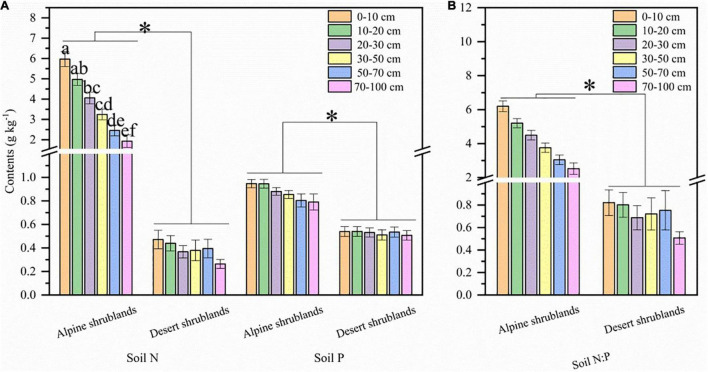
Soil nitrogen (N) content, soil phosphorus (P) content **(A)**, and soil N:P ratios **(B)** in different soil depths of 0–10, 10–20, 20–30, 30–50, 50–70, and 70–100 cm. Both “*” and different lowercase letters indicate significant differences at the level of *P* < 0.05. The results are shown as mean values ± standard error.

Spatially, soil N:P ratio was generally higher in the south than in the north and lower in the west than in the east in shrublands at a soil depth of 0–10 cm (Figure 2A), similar to soil depths of 10–20 (Figure 2B), 20–30 (Figure 2C), 30–50 (Figure 2D), 50–70 (Figure 2E), and 70–100 (Figure 2F) across northeast the Tibetan Plateau. Vertically, with increasing soil depth, the soil N:P ratio had a decreasing trend in alpine shrublands, but this trend was not observed in desert shrublands (Figure 3B). Both soil N and P in alpine shrublands also showed a decreasing trend with soil depth, and this trend was not observed in desert shrublands (Figure 3A). Furthermore, results from variance analysis showed that soil N:P ratios were also higher in alpine shrublands than in desert shrublands in the soil depths of 0–10, 10–20, 20–30, 30–50, 50–70, and 70–100 cm (*P* < 0.05).

**FIGURE 4:**
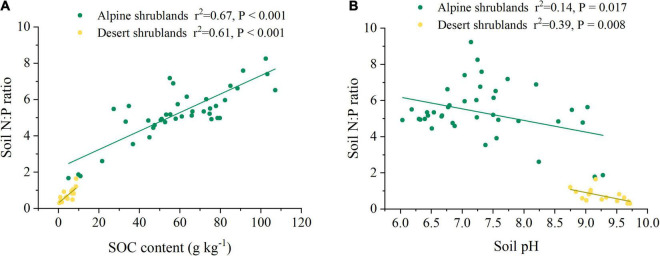
The relationship among soil organic carbon (SOC) **(A)**, soil pH **(B)**, and soil N:P ratio. Yellow circles indicated desert shrublands, while green circles indicate alpine shrublands.

Soil organic carbon (SOC) had a positive effect on soil N:P ratios (Figure 4A). In contrast, the soil N:P ratio showed a decreasing trend with increasing soil pH (Figure 4B). Results from the model of multivariate regressions showed that more than half of the soil N:P ratio variation (*r*^2^ = 0.76 in alpine shrublands and *r*^2^ = 0.89 in desert shrublands) (Table 1) could be explained by soil factors, including soil N, SOC, and soil pH, which indicated that soil characteristics were a robust factor in controlling soil N:P ratios, which supported our hypothesis. In addition, we also quantified their relationship in soil depths of 0–10, 10–20, 20–30, 30–50, 50–70, and 70–100 cm (*P* < 0.05) (Supplementary Table 1).

**TABLE 1:** Model results of multivariate regressions among soil nitrogen: phosphorus (N:P) ratio soil organic carbon (SOC) content, soil N content, and soil pH at 0–100 cm in the Tibetan Plateau shrublands.

Types	Eqn	*r* ^2^	*P*
Alpine shrublands	Soil N:P ratio = 0.61 × Soil N + 0.03 × SOC + 0.65 × Soil pH − 4.12	0.76	< 0.001
Desert shrublands	Soil N:P ratio = 1.32 × Soil N – 0.01 × SOC − 0.04 × Soil pH + 0.56	0.89	< 0.001
Total shrublands	Soil N:P ratio = 0.95 × Soil N + 0.01 × SOC + 0.51 × Soil pH − 4.12	0.90	< 0.001

**FIGURE 5:**
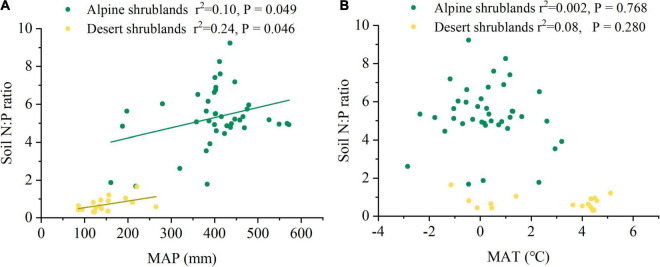
The relationships among mean annual precipitation (MAP) **(A)**, mean annual temperature (MAT) **(B)**, and soil nitrogen: phosphorus (N:P) ratio. Yellow circles indicate desert shrublands, while green circles indicate alpine shrublands.

**FIGURE 6:**
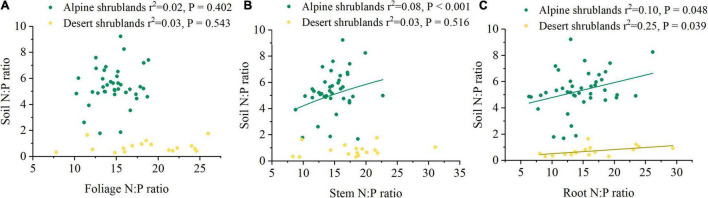
The relationships among nitrogen: phosphorus (N:P) ratio in foliage **(A)**, stem **(B)**, root **(C)**, and soil N:P ratio. Yellow circles indicate desert shrublands, while green circles indicate alpine shrublands.

Mean annual precipitation (MAP) had a positive relationship with the soil N:P ratios in both alpine shrublands and desert shrublands (Figure 5A). In contrast to the effects of MAP, the effects of MAT on soil N:P ratios were marginal, and their negative relationship was not significant (Figure 5B). Shrubland type can also affect the soil N:P ratios (Figure 3). Specifically, the soil N:P ratio in alpine shrublands was larger than that in desert shrublands (*P* < 0.05) (Figure 3). To quantify the effects of the stoichiometry of different organs on soil N:P ratios, we explored the effects of root, stem, and foliage N:P ratios on soil N:P ratios. The root N:P ratios had positive effects on the soil N:P ratios (Figure 6A). The stem N:P ratios, in different shrublands types, had different effects on soil N:P ratios; specifically, stem N:P ratio in alpine shrublands could affect soil N:P ratios, while the effects of stem N:P were marginal in desert shrublands (Figure 6B). Although the soil N:P ratios showed an increasing trend with increasing foliage N:P ratios (Figure 6C), their relationship was not significant (*P* > 0.05).

### Effects of Soil, Climate, and Vegetation Factors on Soil N:P Stoichiometry

**FIGURE 7:**
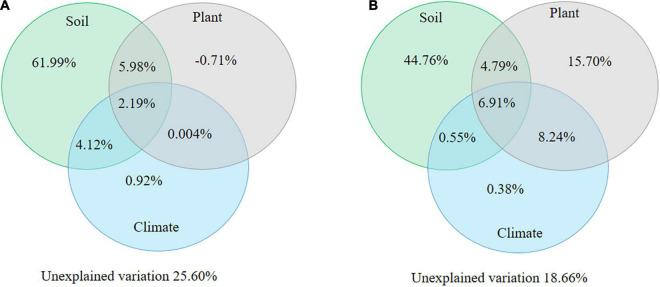
Variation partitioning analysis of soil nitrogen: phosphorus (N:P) ratio in alpine shrublands **(A)** and desert shrublands **(B)** on the northeast Tibetan Plateau. Soil properties include soil N, soil organic carbon, and soil pH. Vegetation properties refer to stem N:P ratios and root N:P ratio alpine shrublands, root N:P ratio in desert shrublands, and shrublands types in all shrublands. Climate refers to mean annual precipitation. Variation partitioning analysis consists of explained variation, including the effects from the soil, climate, vegetation properties, and their combined effects, and unexplained variation.

Furthermore, the variables of SOC, soil pH, and soil N significantly contributed to the soil N:P ratios and were regarded as qualitative variables of soil properties. Plant properties referred to root N:P ratio in both alpine shrublands and desert shrublands, and climate factors referred to MAP. The results of the variation partitioning analysis demonstrated that a significant portion of the variation in the soil N:P ratios was explained by the aforementioned factors, including soil, climate, and plant properties (Figure 7). The explained variation in the N:P ratio was 74.40% in alpine shrublands (Figure 7A) and 81.34% in desert shrublands (Figure 7B). Of these, the effect from soil properties *per se* was the largest in alpine shrublands and accounted for 61.99% of the variation in the N:P ratio (Figure 7A). Similar to desert shrublands, the largest explained variation was also from soil properties *per se* and could be as high as 44.76% (Figure 7B).

**FIGURE 8:**
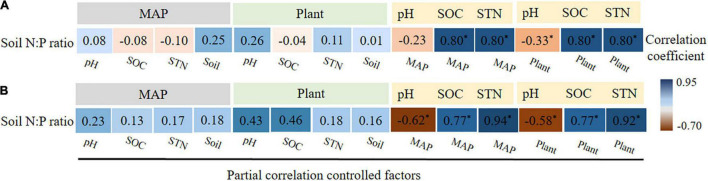
Partial correlations between controlling factors, such as soil properties, plants, mean annual precipitation (MAP), and soil nitrogen (N): phosphorus (P) ratio in alpine shrublands **(A)** and desert shrublands **(B)**. Plant factor refers to root N:P ratio. “*” indicates a significant difference at the level of *P* < 0.05.

Partial correlation analysis indicated that soil properties, including soil pH, SOC, and soil N, were considered as partial correlation-controlled factors, and the relationship between climate factors and soil N:P ratios was insignificant (Figures 8A,B). Similar to plant factors, when soil properties were considered as partial correlation-controlled factors, the relationship between plant factors and soil N:P ratios was also insignificant in alpine shrublands and desert shrublands (Figure 8). However, when climatic factors or plant factors were considered as partially controlled factors, soil properties still had significant effects on the soil N:P ratios (*P* < 0.05) (Figure 8). These results demonstrate that soil properties play a significant role in regulating the relationship between climate factors and soil N:P ratios, and plant factors and soil N:P ratios.

**FIGURE 9:**
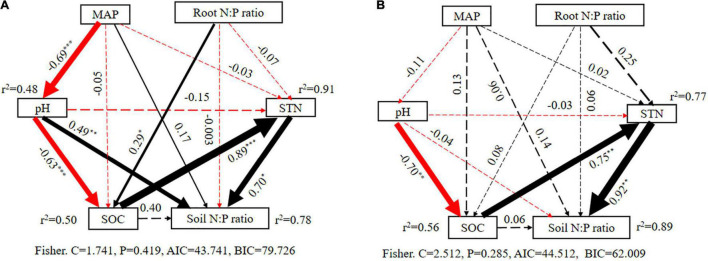
Structural equation models illustrating the effects from direct and indirect factors on soil nitrogen (N):phosphorus (P) ratio in alpine shrublands **(A)** and desert shrublands **(B)**. The width and values of the arrow indicate the relative effect size and standardized path coefficients. The dashed arrows show pathways of non-significant effects (*P* > 0.05). MAP: mean annual precipitation, SOC: soil organic carbon, STN, soil total nitrogen. “*”, “**”, and “***” indicate significant differences at the levels of *P* < 0.05, *P* < 0.01, and *P* < 0.001.

Furthermore, structural equation modeling also showed a similar result from variation partitioning analysis and partial correlation analysis. Specifically, all factors could explain 78% in alpine shrublands and 89% in desert shrublands of the variation in the N:P ratio (Figure 9), which was similar to 74 and 81% with variation partitioning analysis. Furthermore, the standardized effect path coefficient could be as high as 0.70 and 0.92 in alpine shrublands and desert shrublands, which highlighted the significant role of soil factors in shaping soil N:P ratios (Figure 9). Although the root N:P ratio was significantly related to soil N:P ratios in the single variable model (Figure 6), it did not exert significant effects on soil N:P ratios in structural equation modeling in alpine shrublands (Figure 9). Similar to MAP and soil pH in desert shrublands, they did not indicate significant roles in the final structural equation model (Figure 9).

## Discussion

### The Dominant Role of Edaphic Variables in Determining Soil N:P Stoichiometry

The soil N:P ratios showed an increasing trend with increasing SOC (Figure 4) similar to soil N content and soil P content, which also showed an increasing trend with SOC (Supplementary Figure 2). Soil organic matter is considered the main source of soil N (Nie et al., 2017), while it is also a partial source of soil P (Delgado-Baquerizo et al., 2013). Therefore, both soil N content and soil P content showed an increasing trend with SOC, and soil N content increased more than soil P content. Consequently, the soil N:P ratio had an increasing trend with SOC.

There was a significant decrease in the soil N:P ratio with increasing soil pH in both alpine shrublands and desert shrublands (Figure 4). Soil pH can affect both soil N content and soil P content (Supplementary Figure 3), and the decrease in soil N was larger than soil P content, consequently resulting in a decreasing trend with increasing soil pH. The relationship between soil N:P ratio and soil pH in shrublands was similar to the results of negative relationships in the alpine steppe on the Tibetan Plateau (Zhang et al., 2019b). First, soil pH plays a significant role in shaping the microbial composition and microbial diversity (Zhalnina et al., 2015) and plays an important role in driving microbial activities. Thus, it can affect soil organic matter solubility and stabilization (Zolnik and Burgess, 2007), for example, stimulating soil microorganism activity contributes to the decomposition of soil organic matter under moderate alkaline conditions (Whittinghill and Hobbie, 2012). Second, reducing solubility and deficiencies elements, such as zinc manganese and iron, in alkaline soil conditions can limit the growth of plants (Grybos et al., 2009), thereby limiting litter input to soil and resulting in the slow accumulation of soil organic matter (Wu et al., 2017). Therefore, soil N and soil P showed a decreasing trend with increasing soil pH.

It has been proven that the deposition of nitrogen has also been existing on the Tibetan Plateau (Lü and Tian, 2007). Furthermore, the deposition of nitrogen contributes to a decrease in soil pH (Yang et al., 2015), which contributes to an increase in the soil N:P ratio. It has been demonstrated that decreasing soil pH can increase SOC (Nie et al., 2019), which also contributes to an increase in the soil N:P ratio.

### Effects of Climatic Factors on Soil N:P Stoichiometry

As precipitation increased, the soil N:P ratio in both alpine shrublands and desert shrublands showed an increasing trend (Figure 5). However, the driving factors of the positive relationship between soil N:P ratio and MAP were different in alpine and desert shrublands. Specifically, in alpine shrublands, both soil N and soil P content significantly increased with precipitation (Supplementary Figure 4), and the increase in soil N was larger than soil P content, which resulted in a positive relationship between soil N:P ratio and MAP. However, in desert shrublands, the effects of MAP on soil N and soil P were marginal, and soil N showed a slightly increasing trend, whereas soil P showed a decreasing trend (Supplementary Figure 4), resulting in an increasing trend between soil N:P ratio and MAP in desert shrublands.

The positive relationship between soil N:P ratio and MAP was different from previous findings derived from forest soils of China, which showed a decreasing trend between MAP and soil N:P ratio (Chen et al., 2016b). However, our result was similar to the findings from soils in arid areas, which tend to have lower N:P ratios with increasing degrees of aridity (Delgado-Baquerizo et al., 2013). Our results highlighted the diverse patterns of soil N:P ratios along with precipitation. The divergent effects of precipitation on soil N:P ratios between forests in China and our results could be ascribed to the different effects of precipitation on nutrient supply in different biomes (Chen et al., 2016b). Aridity is one of the most distinct climatic conditions of the Tibetan Plateau (Nie et al., 2018), and climate controls on biogeochemical cycles were particularly correlative with arid climates due to biological activity driven by water availability (Schwinning and Sala, 2004). Thus, more precipitation can stimulate biological activities, including microbial mineralization, atmospheric N fixation, photosynthesis (Vicente-Serrano et al., 2012), and plant growth, which contribute to organic matter accumulation (Yang et al., 2008; Nie et al., 2018). Thus, an increase in precipitation will enhance soil N inputs by stimulating the activity of the community (Delgado-Baquerizo et al., 2013). Therefore, increasing precipitation can increase soil N content in arid environments.

Compared with soil N content, the effects of precipitation on soil P content were more complex in the northeast Tibetan Plateau shrublands. Specifically, soil P content showed a significant increasing trend with increasing precipitation in alpine shrublands, while it showed a decreasing trend with increasing precipitation in desert shrublands (Supplementary Figure 4B). To some extent, soil P is derived from the decomposition of organic matter (Delgado-Baquerizo et al., 2013). In alpine shrublands, increasing precipitation can increase organic matter (Nie et al., 2019), thereby increasing soil P content. However, it should be noted that increasing precipitation can also lead to nutrient leaching from soils (Peter et al., 2010; Chen et al., 2016b). It has been suggested that precipitation in forest ecosystems in China can cause relatively more leaching of nutrients, such as soil P, and result in lower availability of these elements in surface soils (Peter et al., 2010; Tian et al., 2010; Chen et al., 2016b). Increasing precipitation can result in a decreasing trend between soil P content and MAP in desert shrublands. The effects of soil P accumulation were more dominant in alpine shrublands, while depletion of nutrients from leaching was more dominant in desert shrublands.

Our results revealed that soil N:P ratios had a significant relationship with MAP, and were relatively stable with increasing MAT (Figure 5) (*P* > 0.05), which suggested that soil N and soil P were more tightly stable along temperature gradients than precipitation gradients. Our results were different from those of a negative relationship in the alpine steppe in the Tibetan Plateau (Zhang et al., 2019b) and the grasslands in the Inner Mongolian Plateau (Jiao et al., 2016). However, the relationship between soil N:P ratio and temperature in Tibetan Plateau shrublands was similar to the results of forests in China (Chen et al., 2016b). Although their relationships were similar, the driving factors for their relationships were different. In China, the soil N:P ratio was relatively stable with temperature, due to the similar decrease in soil N and soil P with increasing temperature (Chen et al., 2016b). However, in the Tibetan Plateau shrublands, soil N and soil P were stable with increasing temperature (Supplementary Figure 5) (*P* > 0.05). Cold climate is another significant climatic character (Ding et al., 2017) and increasing temperature on one hand can stimulate microbial decomposition activity, which can result in a decrease in organic soil matter (Yang et al., 2008). On the other hand, the increasing temperature can contribute to plant growth and shrubland biomass accumulation on the Tibetan Plateau (Nie et al., 2018), which contributes to increased input of soil organic matter and thereby increase soil N and P content (Delgado-Baquerizo et al., 2013). Their effects may be roughly offset, resulting in a stable relationship in the shrublands on the northeast Tibetan Plateau.

### Effects of Plants on Soil N:P Stoichiometry

The effects of plant stoichiometric ratios in different shrubland types on soil stoichiometry were not always similar (Tian et al., 2019). Specifically, the N:P ratio in the stem had significant effects on the soil N:P ratios in alpine shrublands, whereas in desert shrublands, its effects were marginal, which indicated that the shrubland type probably shaped soil N:P ratios through differences in the effects of organs on soil N:P ratios. The relationships were different among the shrubland organ ratios, including foliage and root and soil N:P ratios. Specifically, the N:P ratio in roots can significantly affect the soil N:P ratio, while the effects of foliage N:P ratios on soil N:P ratios were marginal, which indicated that compared with foliage, roots had more significant effects on soil N:P ratios. In subtropical forests, the N:P ratios in the root of the tree also showed a significant increasing trend with soil N:P ratios (Luo et al., 2020), which was similar to our results. However, although the foliage N:P ratios in forests also significantly affected the soil N:P ratios (Luo et al., 2020), their relationship was not significant in shrublands on the northeast Tibetan Plateau. Compared with aboveground biomass, roots are more efficient in shaping the N:P ratio. There are several reasons for this. First, roots promote the aggregation of mineral particles, which contribute to soil organic matter formation through their chemical composition (Lange et al., 2015; Rumpel and Chabbi, 2019). Both root exudates and dissolved organic carbon play important roles in shaping soil N and P content (Schmidt et al., 2011; Luo et al., 2020). Second, roots are distributed more closely to soil minerals and litter deposited across different soil layers (Rasse et al., 2005; Rumpel and Chabbi, 2019). It should be noted that plants are associated with beneficial organisms, including growth-promoting bacteria and rhizobia, which potentially shape P and N cycles on the root surface (Nuccio et al., 2013). Third, plant uptake may lead to a decrease in the availability of mineral nutrients for soil microorganisms (Schimel et al., 1989), and affect microbial communities during the decomposition of organic matter (Rumpel and Chabbi, 2019). Therefore, roots were more important in shaping soil stoichiometry in the shrublands on the northeast Tibetan Plateau.

## Conclusion

We quantified the distribution of soil N:P ratio and its controlling factors among edaphic, plant, and climate factors in the northeast Tibetan Plateau shrublands. Spatially, the soil N:P ratio was higher in the south than in the north and lower in the west than in the east. The soil N:P ratio in the northeast Tibetan Plateau shrublands was mainly explained by edaphic factors, which also played an important role in regulating the effects of plant and climate factors on the soil N:P ratios. MAP, rather than MAT, significantly controlled the soil N:P ratio. Different shrubland organ N:P ratios also played different roles in shaping the soil N:P ratio in alpine shrublands and desert shrublands across the northeast Tibetan Plateau. Our study contributes to a deeper understanding of biogeochemical cycles at high altitudes.

## Data Availability Statement

All data supporting the findings of the study are available on request from the corresponding authors.

## Author Contributions

GZ and DW designed the experiments. GZ, LY, YC, LR, KM, and XN performed the experiments and collected the data. XN and GZ analyzed the data. XN, GZ, YD, and DW wrote the article. All authors contributed to the article and approved the submitted version.

## Conflict of Interest

The authors declare that the research was conducted in the absence of any commercial or financial relationships that could be construed as a potential conflict of interest.

## Publisher’s Note

All claims expressed in this article are solely those of the authors and do not necessarily represent those of their affiliated organizations, or those of the publisher, the editors and the reviewers. Any product that may be evaluated in this article, or claim that may be made by its manufacturer, is not guaranteed or endorsed by the publisher.

## Footnotes

1
http://www.worldclim.org/
