# Supplementary figures and images for: Distribution Characteristics and Controlling Factors of Soil Total Nitrogen: Phosphorus Ratio Across the Northeast Tibetan Plateau Shrublands
Source: Front Plant Sci. 2022 Apr 12;13:825817. doi: 10.3389/fpls.2022.825817 (PMC9039665; doi:10.3389/fpls.2022.825817)

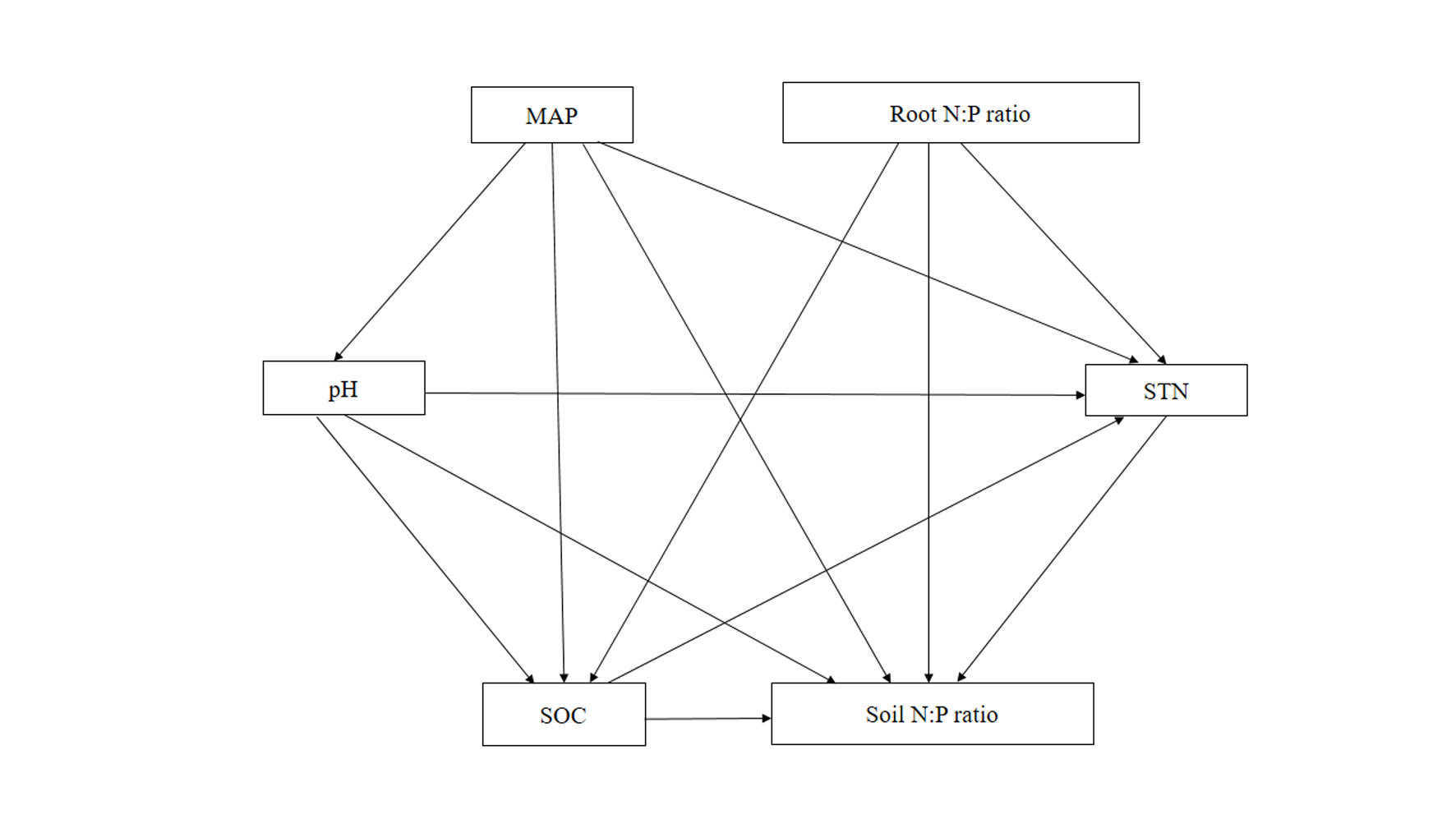

Supplement: Supplementary file 1 [file Image_1.TIF]
