# Supplementary material for: Distribution Characteristics and Controlling Factors of Soil Total Nitrogen: Phosphorus Ratio Across the Northeast Tibetan Plateau Shrublands
Source: Front Plant Sci. 2022 Apr 12;13:825817. doi: 10.3389/fpls.2022.825817 (PMC9039665; doi:10.3389/fpls.2022.825817)
Supplement: Supplementary file 6 [file Table_1.docx]

**Table S1.** Model results of multivariate regressions among soil nitrogen: phosphorus (N:P) ratio soil organic carbon (SOC) content, soil N content, and soil pH in the different soil depths in the northeast Tibetan Plateau shrublands.

| Soil depths (cm) | Eqn | | r^2^ | P |
| --- | --- | --- | --- | --- |
| 0-10 | | Soil N:P ratio = 0.97 × Soil N + 0.002 × SOC + 0.34 × Soil pH − 2.36 | 0.87 | < 0.001 |
| 10-20 | | Soil N:P ratio = 1.55 × Soil N - 0.02 × SOC - 0.42 × Soil pH + 2.69 | 0.96 | < 0.001 |
| 20-30 | | Soil N:P ratio = 1.12 × Soil N - 0.002 × SOC + 0.30 × Soil pH − 2.26 | 0.92 | < 0.001 |
| 30-50 | | Soil N:P ratio = 1.23 × Soil N - 0.02× SOC + 0.04 × Soil pH + 0.04 | 0.91 | < 0.001 |
| 50-70 | | Soil N:P ratio = 0.94 × Soil N + 0.004 × SOC - 0.09 × Soil pH + 1.33 | 0.90 | < 0.001 |
| 70-100 | | Soil N:P ratio = 1.55 × Soil N - 0.03 × SOC + 0.01 × Soil pH + 0.20 | 0.92 | < 0.001 |
